# Correlation Between Bladder Pain Syndrome/Interstitial Cystitis and Pelvic Inflammatory Disease

**DOI:** 10.1097/MD.0000000000001878

**Published:** 2015-11-20

**Authors:** Shiu-Dong Chung, Chao-Hsiang Chang, Peir-Haur Hung, Chi-Jung Chung, Chih-Hsin Muo, Chao-Yuan Huang

**Affiliations:** From the Division of Urology, Department of Surgery, Far Eastern Memorial Hospital, New Taipei City (S-DC); School of Medicine, College of Medicine, Fu-Jen Catholic University, New Taipei (S-DC); Graduate Program in Biomedical Informatics, College of Informatics, Yuan Ze University (S-DC); Department of Urology, China Medical University and Hospital (C-HC); Department of Medicine, College of Medicine, China Medical University and Hospital, Taichung (C-HC, C-HM); Department of Internal Medicine, Ditmanson Medical Foundation Chiayi Christian Hospital, Chiayi (P-HH); Department of Applied Life Science and Health, Chia-Nan University of Pharmacy and Science, Tainan (P-HH); Department of Health Risk Management, College of Public Health, China Medical University (C-JC); Department of Medical Research, China Medical University and Hospital (C-JC); Management Office for Health Data, China Medical University and Hospital, Taichung (C-HM); and Department of Urology, National Taiwan University Hospital, College of Medicine, National Taiwan University, Taipei, Taiwan (C-YH).

## Abstract

Pelvic inflammatory disease (PID) has been investigated in Western countries and identified to be associated with chronic pelvic pain and inflammation. Bladder pain syndrome/interstitial cystitis (BPS/IC) is a complex syndrome that is significantly more prevalent in women than in men. Chronic pelvic pain is a main symptom of BPS/IC, and chronic inflammation is a major etiology of BPS/IC. This study aimed to investigate the correlation between BPS/IC and PID using a population-based dataset.

We constructed a case–control study from the Taiwan National Health Insurance program. The case cohort comprised 449 patients with BPS/IC, and 1796 randomly selected subjects (about 1:4 matching) were used as controls. A Multivariate logistic regression model was constructed to estimate the association between BPS/IC and PID.

Of the 2245 sampled subjects, a significant difference was observed in the prevalence of PID between BPS/IC cases and controls (41.7% vs 15.4%, *P* < 0.001). Multivariate logistic regression analysis revealed that the odds ratio (OR) for PID among cases was 3.69 (95% confidence interval [CI]: 2.89–4.71). Furthermore, the ORs for PID among BPS/IC cases were 4.52 (95% CI: 2.55–8.01), 4.31 (95% CI: 2.91–6.38), 3.00 (95% CI: 1.82–4.94), and 5.35 (95% CI: 1.88–15.20) in the <35, 35–49, 50–64, and >65 years age groups, respectively, after adjusting for geographic region, irritable bowel syndrome, and hypertension. Joint effect was also noted, specifically when patients had both PID and irritable bowel disease with OR of 10.5 (95% CI: 4.88–22.50).

This study demonstrated a correlation between PID and BPS/IC. Clinicians treating women with PID should be alert to BPS/IC-related symptoms in the population.

## INTRODUCTION

Pelvic inflammatory disease (PID) is a common gynecologic disorder in women ages <45 years old. PID is commonly manifested as acute abdominal pain, and it is associated with tubo-ovarian abscesses requiring aggressive antimicrobial therapy and radiological intervention. In the United States, about 800,000 women have experienced at least 1 episode of PID.^1^ This upper reproductive tract infection is usually caused by a sexually transmitted infection and will increase the risk for tubal infertility, ectopic pregnancy, and chronic pelvic pain, which can impair the quality of life in women.^2^

Bladder pain syndrome/interstitial cystitis (BPS/IC) is a urologic disease that is difficult to manage and characterized by pelvic pain, urinary urgency, frequency, and nocturia.^3,4^

The quality of life for patients with BPS/IC might be impaired because of chronic pelvic pain and irritating voiding or storage urinary symptoms. BPS/IC and PID share pelvic pain as a common symptom, and both are associated with chronic inflammation. Nevertheless, no study to date has reported the association between PID and BPS/IC. In the present investigation, we conducted a large scale, controlled cohort study in Taiwan to examine the correlation between BPS/IC and PID.

## MATERIALS AND METHODS

### Data Source

Taiwan Bureau of National Health Insurance (NHI) established a National Health Insurance program on March 1, 1995. All Taiwan residents compulsorily joined this program, which had an insurance coverage ratio of over 99% in 2010. We obtained a Longitudinal Health Insurance (LHID) database for this case–control study. LHID contained 1 million insurants randomly selected from the original registry for beneficiaries during 1996 to 2000. LHID included all medical claims and treatment records for each insurant from 1996 to 2010. To prevent researchers from attempting to identify a particular patient, the identification of insurants was scrambled. According to the Personal Information Protection Act, this study was approved by the Institutional Review Board of China Medical University Hospital. In LHID, diseases were identified based on the International Classification of Diseases, 9th Revision, Clinical Modification (ICD-9-CM).

### Study Subjects

We collected 449 women with newly diagnosed BPS/IC (ICD-9-CM 595.1X) during 2000 to 2010 as the BPS/IC group. The date for BPS/IC diagnosis was defined as the index date. Four controls were selected from women without BPS/IC history in LHID. Controls were frequency matched with age (±5-year strum), year of entry in the insurance program, and year of index date.

### Interesting Variable

The interesting variables included demographic characteristic and comorbidity. Demographic characteristics comprised age, geographic region (northern, central, southern, and eastern), and occupation (white collar, blue collar, and others). Premium-based income was grouped into 3 levels: <528, 528 to 640, and more than 640 USD (<15,840; 15,840–19,200; and ≥19,200 New Taiwan Dollar, respectively) (1 USD = 30 NTD). Blue-collar workers were identified as individuals with long outdoor work hours, such as farmers, fishermen, and industrial laborers. Meanwhile, white-collar workers were identified as individuals with long indoor work hours, such as government officials, institution workers, businessmen, and industrial administrators. Other occupations included individuals who were retired, unemployed, and had low income. Comorbidity included PID (ICD-9-CM 614.XX), hypertension (ICD-9-CM 401–405), irritable bowel syndrome (IBS, ICD-9-CM 558.91, 564.0X, 564.1X, and 564.5X), inflammatory bowel disease (IBD, ICD-9-CM 555.XX and 556.XX), lupus (ICD-9-CM 710.0X), rheumatic arthritis (ICD-9-CM 714.XX), and abortion-associated disease (ICD-9-CM 630–639). We also categorized pregnancy history into 0, 1, and ≥2. All comorbidities were defined before the index date.

### Statistical Analysis

Chi-squared test was used to test the difference in geographic region, occupation, monthly income level, and comorbidity between the BPS/IC and non-BPS/IC groups. When the number of cells was <5, we used Fisher's exact test to differentiate between the 2 groups. *t* test was used for the mean age difference between the 2 groups. Odds ratio (OR) and 95% confidence interval (CI) of BPS/IC in PID women compared with non-PID women were used in logistic regression. Multivariable model was adjusted for the interesting variables, which displayed a significant difference (Table 1). The association between IC and PID stratified by age was also assessed, and the joint effect for IC and IC-associated risk factor was estimated. SAS (version 9.3 for Windows; SAS Institute, Inc., Cary, NC) was used to perform all statistical analyses. The level of significance was defined as a two-sided *P* value <0.05.

**TABLE 1:**
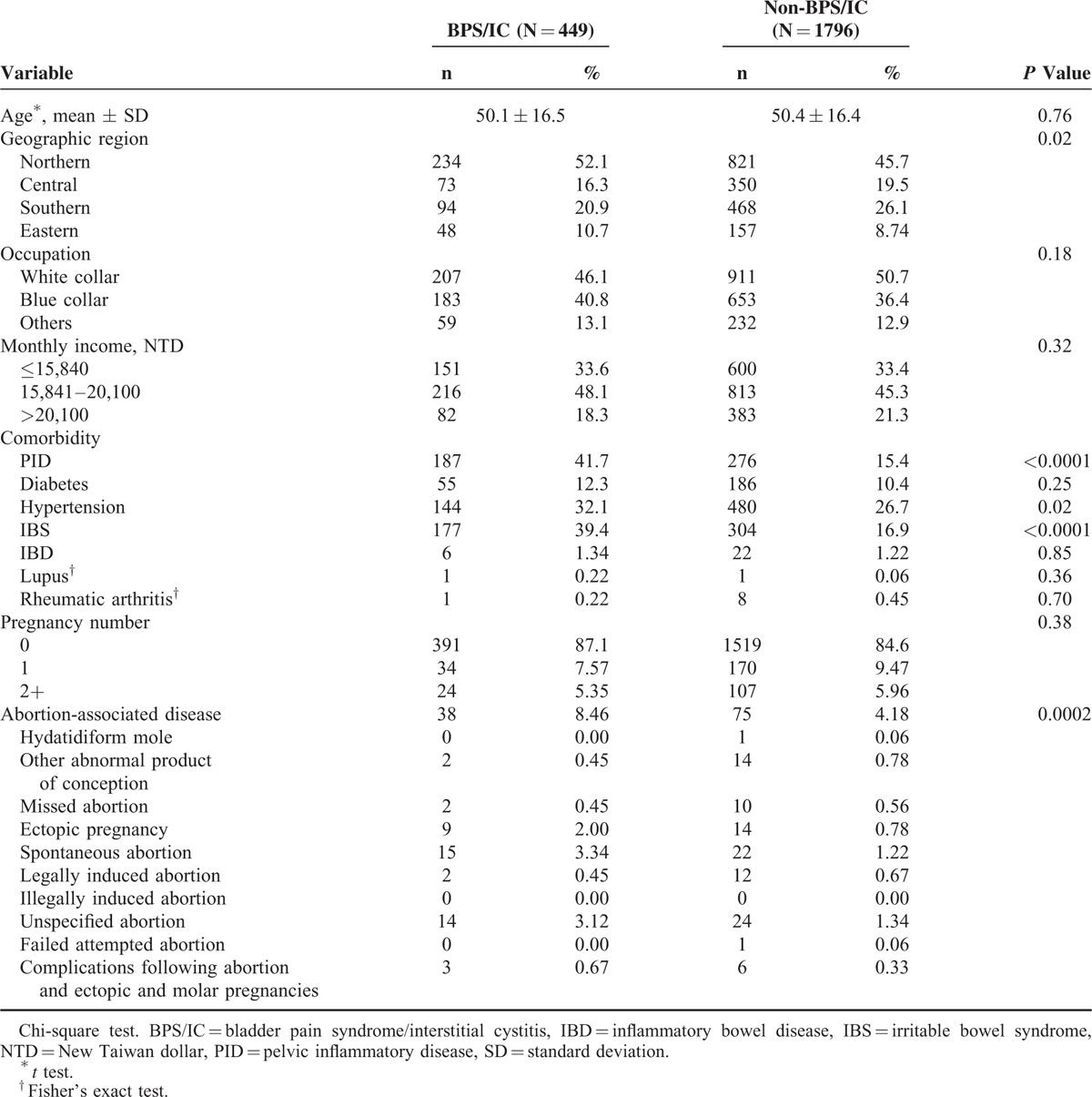
Demographic and Comorbidity Between Patients With and Without BPS/IC

## RESULT

We collected a total of 449 BPS/IC patients and 1796 non-BPS/IC as controls (Table 1). No significant difference was observed for age, occupation, and monthly income between the BPS/IC and non-BPS/IC patients. Compared with the controls, the BPS/IC patients exhibited more cases of PID (41.7% vs 15.4%), hypertension (32.1% vs 26.7%), IBS (39.4% vs 16.9%), and abortion-associated disease (8.46% vs 4.18%). Other comorbidities, including diabetes, IBD, lupus, rheumatic arthritis, and pregnancy numbers, did not show significant differences between the BPS/IC and non-BPS/IC cohorts.

During the study period of 10 follow-up visits, the incidence for BPS/IC in the PID cohort was 3.97-fold higher than that in the non-PID cohort (95% CI: 3.16–4.99) (Table 2). After controlling geographic region, hypertension, and IBS, the PID cohort had 3.69-fold risks of BPS/IC higher than the controls (95% CI: 2.89–4.71). A similar increased risk was observed for the stratification of age (<35, 35–49, 50–64, and ≥65) (both *P* values of <0.05). Moreover, PID (OR: 3.69; 95% CI: 2.89–4.71), hypertension (OR: 1.45; 95% CI: 1.13–1.86), and IBS (OR: 2.69; 95% CI: 2.11–3.42) exerted significant effects on BPS/IC risk, but not abortion-associated disease (OR: 1.35; 95% CI: 0.86–2.11) after adjusting for geographic region and other risk factors. Therefore, we used PID, hypertension, and IBS to analyze the joint effect.

**TABLE 2:**
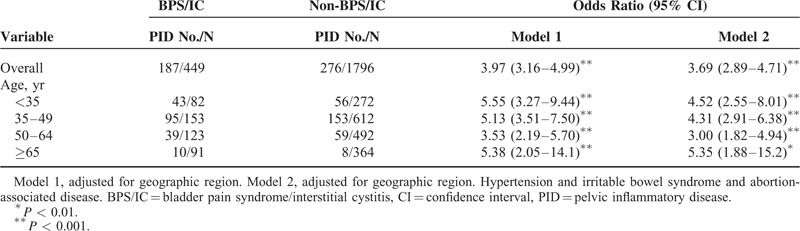
Odds Ratio and 95% Confidence Interval of BPS/IC Risk for PID Participants Compared With Non-PID Participants

Table 3 presents the joint effect of PID and other comorbidities for BPS/IC risk. The highest risk of BPS/IC was in those with PID (OR: 4.33; 95% CI: 3.06–6.12), followed by IBS (OR: 3.64; 95% CI: 2.39–5.54) and hypertension (OR: 1.77; 95% CI: 1.26–2.51). Patients with both PID and hypertension or PID and IBS had 4.32- and 9.92-fold risks of BPS/IC, respectively, compared with those without any PID and other comorbidities. Finally, individuals with PID, hypertension, and IBS had significantly increased risk of BPS/IC (OR: 10.5; 95% CI: 4.88–22.50).

**TABLE 3:**
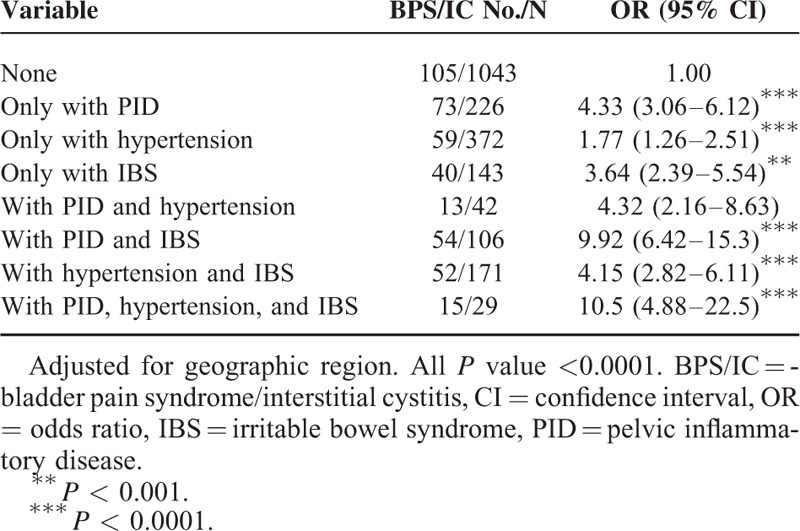
Join Effect for PID and Other Comorbidities for BPS/IC Risk

## DISCUSSION

Our results suggested that the risk of BPS/IC in women with PID was significantly higher than that in women without PID after adjusting for potential confounding factors. To the best of our knowledge, this study is the first regional population-based study to investigate the association between PID and subsequent BPS/IC in an Asian population. In this study, we also found that different age groups of women with PID history were more likely to have BPS/IC than those without PID after adjusting for geographic region, hypertension, and IBS. Moreover, patients with PID, hypertension, and IBS were at high risk of BPS/IC.

Previous studies have reported associations of BPS/IC with nonbladder syndrome, including fibromyalgia, chronic fatigue syndrome, and IBS.^5,6^ They suggested that sympathetic dysfunction may be their common underlying pathogenesis.^6^ Increasing evidence has shown that chronic inflammation is important in the development of BPS/IC, specifically in cases of high severity.^7,8^ A recent study reported that BPS/IC patients have reduced pain thresholds.^9^ Schrepf et al^8^ indicated that Toll-like receptor-4 (TLR-4)-mediated inflammation plays a critical role in pelvic pain of BPS/IC. Previous studies have shown that proinflammatory TLR-4 activation of spinal cord glial cells is essential in the development and maintenance of chronic pain.^10,11^

Another study also demonstrated that experimentally induced systemic inflammation can reduce pain thresholds in healthy subjects.^11^ Therefore, chronic pelvic-localized inflammatory status might be associated with BPS/IC. PID is generally polymicrobial in nature, because multiple bacteria have been isolated from the upper genital tract in women with PID.^12^*Neisseria gonorrhoeae*, *Chlamydia trichomatis*, and *Mycoplasma genitalium* are common pathogens in PID patients.^13–15^ Infection with *M genitalium* has been associated with cervicitis, PID, and tubal factor infertility. McGowin et al^16^ demonstrated that *M genitalium* can result in persistent infection of human endocervical epithelial cells that results in chronic inflammatory cytokine secretion and increased responsiveness to secondary TLR stimulation. TLR-4 stimulation, specifically in spinal glial cells, has been shown to lead to amplified ascending pain signaling via the release of proinflammatory cytokines.

*Mycoplasma genitalium*, a prevalent pathogen in women with PID, can cause persistent human infections associated with chronic reproductive tract inflammation. Ustinova et al^17^ demonstrated that colonic irritation results in bladder inflammation in rats, which implied the existence of neural cross talk between visceral organs in response to noxious stimuli.^18^ Thus, the bladder is more vulnerable to cross-organ inflammation than the colon or uterus. Neuronal cross talk between visceral organs is unidirectional, and it is only propagated from the colon or uterus to the bladder.^19^

Although the paper has 1 major strength that it has the large sample size which can afford statistical advantage in detecting the association between PID and BPS/IC. However, the present study had several limitations. We collected data based on the diagnoses of PID or BPS/IC made by physicians according to the ICD-9-CM codes released by the Bureau of NHI. It is cannot be avoided that some patients were not coded accurately and we might miss those who did not seek medical care. In addition, the severity of PID or BPS/IC from the database is not available. We either were unable to evaluate whether severity or frequency of PID will be associated with higher risk of BPS/IC than those with mild or less frequent PID. Previous pelvic operations or trauma history might also be associated with the occurrence of BPS/IC symptoms; however, these data were absent. Furthermore, some patient information on factors such as family history, tobacco use, body weight, sleep condition, bowel habits which may have had an effect on BPS/IC was not available through the database.

Despite these limitations, this study provides epidemiological evidence of a link between PID and subsequent BPS/IC diagnosis. The specific mechanisms underlying this relationship remain unclear. Further study is needed to confirm these findings and explore the underlying pathomechanisms. In addition, clinical practitioners treating subjects with PID should be reminded to be alert for urinary complaints.

In summary, our study demonstrated a correlation and epidemiological evidence of a link between PID and BPS/IC. Clinicians treating female subjects with PID should alert them about the correlation between PID and BPS/IC.
